# Multilocus Genotyping of ‘*Candidatus* Phytoplasma solani’ Associated with Rubbery Taproot Disease of Sugar Beet in the Pannonian Plain

**DOI:** 10.3390/microorganisms9091950

**Published:** 2021-09-14

**Authors:** Živko Ćurčić, Andrea Kosovac, Jelena Stepanović, Emil Rekanović, Michael Kube, Bojan Duduk

**Affiliations:** 1 Institute of Field and Vegetable Crops, 21000 Novi Sad, Serbia; zivko.curcic@gmail.com; 2 Institute of Pesticides and Environmental Protection, 11080 Belgrade, Serbia; andrea.kosovac@pesting.org.rs (A.K.); jelena.stepanovic@pesting.org.rs (J.S.); emil.rekanovic@pesting.org.rs (E.R.); 3 Department of Integrative Infection Biology Crops-Livestock, University of Hohenheim, 70599 Stuttgart, Germany; michael.kube@uni-hohenheim.de

**Keywords:** sugar beet, red beet, stolbur phytoplasma, ‘*Candidatus* Phytoplasma solani’, RTD, SBR, MLSA, Serbia

## Abstract

Rubbery taproot disease of sugar beet (RTD), associated with ‘*Candidatus* Phytoplasma solani’, appeared in 2020 on an epidemic scale in northern Serbia and southern Slovakia, situated at opposite edges of the Pannonian Plain. In the affected locations where the disease was assessed, symptomatic sugar beets were analysed for phytoplasma infection. Additionally, multilocus sequence analyses of ‘*Ca*. P. solani’ strains on epidemiologically informative marker genes (*tuf*, *stamp* and *vmp1*) were performed. Symptomatic sugar beets from other countries of the Pannonian Plain (Croatia, Hungary and Austria), one sample from Germany, and red beets from Serbia were included in the analyses. ‘*Ca*. P. solani’ was detected in sugar beet in all assessed countries, as well as in red beet. Molecular analyses revealed the high genetic variability of ‘*Ca*. P. solani’ with the presence of all four *tuf-*types (a, b1, b2 and d), 14 *stamp* genotypes (seven new) and five *vmp1* profiles (one new). The most common multilocus genotype in Serbia, Slovakia, Croatia, and Hungary was dSTOLg (tuf-d/STOL/V2-TA). It was dominant on sites with epidemic RTD outbreaks in the Pannonian Plain and in several sugar beet fields with non-epidemic RTD occurrence suggesting the prevalence of a particular epidemiological pathway during the epidemic’s phases.

## 1. Introduction

Phytoplasmas are intracellular, phloem-limited plant pathogenic bacteria belonging to the class *Mollicutes*. These biotrophic, wall-less prokaryotes are transmitted via phloem-feeding insect vectors. Initially, phytoplasmas were delineated to 16Sr groups on the basis of the RFLP analysis of the 16S rRNA gene [1]. Sequence analysis of the 16S rRNA gene classified this pathogen within the provisional genus ‘*Candidatus* Phytoplasma’, currently accounting for more than 40 formally described taxons [2].

‘*Candidatus* Phytoplasma solani’ (stolbur phytoplasma, 16SrXII-A subgroup), is an obligate plant pathogen that infects a wide range of plant species, occasionally inducing severe economic losses on several crops throughout Europe [3,4]. A wide host range, both among crops and wild plants, coupled with several insect vectors of the suborder Auchenorrhyncha (Hemiptera) with a broad feeding range, contribute to the complexity of stolbur phytoplasma biology [5,6,7]. ‘*Ca*. P. solani’ has recently been associated with long-known, but poorly-understood rubbery taproot disease of sugar beet (RTD) in Serbia [8]. Typical symptoms of RTD-affected sugar beets are yellowing and necrosis of the oldest leaves, starting from their margins, while the taproot wilts and becomes rubbery. This rubberiness of the taproot is an RTD symptom of particular economic interest that remains noticeable after harvest and hinders sugar beets from being further processed by the sugar industry.

The most prominent vector of ‘*Ca*. P. solani’ is *Hyalesthes obsoletus*, polyphagous planthopper associated with *Convolvulus arvensis* and *Urtica dioica* in central Europe [9,10]. The genetic variability of ‘*Ca*. P. solani’, based on the conservative house-keeping *tuf* gene (encoding factor tu), is correlated in Europe with the inoculum source plant [10,11,12,13]. Accordingly, a specific ‘*Ca*. P. solani’ reservoir plant could be determined as follows: tuf-a and tuf-b2 types are harboured by *U. dioica*, while tuf-b1 is associated with *C. arvensis*, *Crepis foetida* and *Vitex agnus-castus*, with all four plants simultaneously hosting *H. obsoletus* populations [11,12,13,14]. While the genotypisation of the ‘*Ca*. P. solani’ strains related to other crop diseases in Serbia has revealed the presence of the *tuf* type b1, the recently disclosed aetiology of sugar beet RTD has described the presence of the new *tuf* genotype, designated “tuf-d type” [8]. The newly described *tuf* type was the most commonly found in the RTD-affected sugar beet fields previously assessed in north Serbia [8]. It was also sporadically detected in other crops (parsley and parsnip), but always associated with ‘*Ca.* P. solani’ strains determined as the STOL genotype based on the *stamp* gene (stolbur phytoplasma antigenic membrane protein) [8]. While the ecological and epidemiological significance of the tuf-d type is still to be evaluated, recent studies in Asia (Iran) have boosted ‘*Ca*. P. solani’ strains genotypisation based on this particular gene [15,16]. Molecular characterisation on highly variable genes such as *stamp* and *vmp1* (variable membrane protein), has already been used, not only to access genetic variability, but also to give an insight into the complex epidemiological cycles of these plant pathogenic microorganisms that spread through natural and cultivated vegetation [12,17,18,19].

Another phloem-limited biotroph, namely ‘*Candidatus* Arsenophonus phytopathogenicus’ [20], is associated with “basses richesses” (SBR), sugar beet disease described in western European countries (France, Germany, and Switzerland) [21,22,23]. Typical symptoms of SBR include ones not present in the case of RTD (root vascular tissue discolouration and necrosis and leaf deformation), but also aspecific ones (reduced growth and leaf chlorosis and proliferation). This pathogen has not been detected in Serbia so far, but limited numbers of previously assessed localities could have concealed its presence in the field [8]. However, ‘*Ca*. P. solani’ has been sporadically detected in SBR-affected sugar beets in France, and reported to induce symptoms similar to SBR, albeit with a minor role in the disease aetiology [24]. Moreover, one sugar beet sample has been reported as infected with ‘*Ca*. P. solani’ in southern Russia. In both cases, RFLP analyses of the *tuf* gene have suggested an affiliation of the strains to the tuf-b type [25,26,27].

Since the middle of the 20th century, RTD has been known as a yield-reducing sugar beet disease occurring in Serbia and neighbouring Bulgaria and Romania [28,29,30]. The appearance of RTD-like symptoms was recently observed, at different rates, in all sugar beet-growing areas in northern Serbia and in a sugar beet-growing area in Farna, southern Slovakia-two regions situated at the southern and northern edges of the Pannonian Plain, respectively. This raised concerns about a Pannonian Plain-wide impact of sugar beet RTD and that it could be present in Central Europe. The present study aims to investigate RTD of sugar beet through (I) field surveys for screening and determining the distribution of ‘*Ca*. P. solani’ and ‘*Ca*. A. phytopathogenicus’ in RTD-symptomatic sugar beets in Serbia and across the Pannonian Plain, and (II) multilocus sequence analyses (MLSA) of the RTD-associated microorganism ‘*Ca*. P. solani’, based on informative genetic markers (*tuf*, *stamp* and *vmp1*).

The obtained results extended knowledge on the ‘*Ca*. P. solani’ presence on sugar beet in Serbia covering the entire sugar beet production area situated in the countries’ north. First records of the ‘*Ca*. P. solani’-infected sugar beet in Slovakia, Croatia, Hungary, Austria, and Germany are provided. Red beet in Serbia with RTD-like symptoms was also confirmed for the first time as a ‘*Ca*. P. solani’ host. Genetic diversity of the ‘*Ca*. P. solani’ strains associated with RTD was assessed in sugar beet fields with epidemic and non-epidemic RTD occurrence revealing the presence of the various ‘*Ca*. P. solani’ strains, associated with both known epidemiological cycles, tuf-a/b2, and tuf-b1. Provided MLSA results contribute to the knowledge on the ‘*Ca*. P. solani’ genetic variability through detection of 15 (five new) three-gene multilocus genotypes and seven partially characterised comprehensive genotypes. The multilocus genotype tuf-d/STOL/V2-TA (dSTOLg) is highlighted as the prevailing Ca. P. solani’ strain, possibly involved in the epidemiological outbreaks of the RTD of sugar beet across the Pannonian Plain.

## 2. Materials and Methods

### 2.1. Symptom Observation and Sample Collection

**Figure 1:**
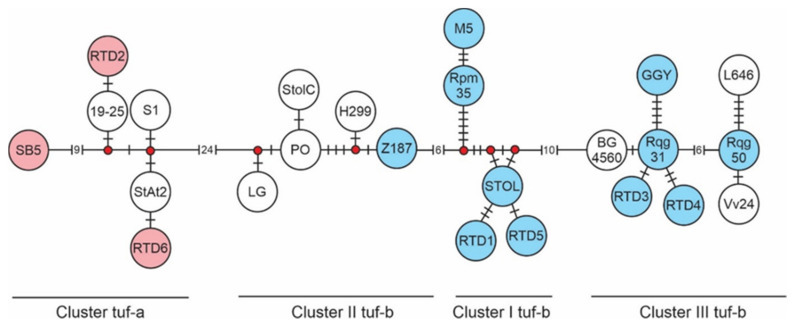
Median-joining network obtained for the *stamp* gene sequences of ‘*Ca.* Phytoplasma solani’ strains detected in sugar beets from Serbia, Slovakia, Hungary, Croatia, Austria and Germany, red beets from Serbia and for reference strains previously detected in Central Europe [11,36,38]. All *stamp* genotypes detected in this study are represented by a circle of a specific colour either red or blue. ‘*Ca*. P. solani’ strains belonging to the cluster tuf-a include samples associated with tuf-a and tuf-b2 types and are represented in red circles, while genotypes in the clusters I, II and III tuf-b, that encompass ‘*Ca*. P. solani’ strains of the tuf-b1 and tuf-d types, are in blue. Circles with no shade represent *stamp* genotypes previously detected in Central Europe and/or Serbia, but not found during this research. Black dashes on the lines connecting genotypes represent the number of mutations; more than five nucleotide differences are given in parenthesis as corresponding number of mutations in-between genotypes. Red interconnecting dots in the network are median vectors that represent missing or unsampled intermediate genotypes.

During October 2020, a survey on sugar beet RTD symptoms was carried out at 11 locations in Serbia and one in Slovakia (Table 1, Figure 1). Additionally, red beets with RTD-like symptoms were sampled from a 10 ha intensive production field at Stepanovićevo, Serbia. Typical RTD symptoms were determined based on a visual assessment of leaves and the flexibility of taproots. Based on the disease incidence, the surveyed localities were divided into those with high or low disease frequencies, i.e., epidemic or non-epidemic RTD occurrence, respectively. In each of the assessed fields, four randomly selected 60 m-long rows, containing approximately 300 sugar beets each, were visually inspected and the number of RTD symptomatic plants was scored. The percentage of symptomatic plants in each field was calculated by averaging the percentage of symptomatic plants in the four rows (Table 1). Disease incidences of ≤0.5% and ≥5% were considered as the thresholds for considering disease frequency in a certain field as low or high, respectively. Since the outbreak of RTD symptoms is usually aggregated in a field, and sugar beet fields are usually large, estimations of disease frequency as low or high were further confirmed in each field as follows. The area with the highest number of symptomatic sugar beet plants was visually selected and set as an estimation plot of 1a (a rectangle 10m × 10m, containing ~1000 plants). Disease frequency on the defined estimation plot was determined by counting and calculating the percentage of symptomatic plants. Disease incidence of ≤2% and ≥20% were set as the thresholds for confirming a certain locality as having a low or high disease frequency, respectively. In the RTD root symptoms assessment, the precipitation data for all localities in Serbia were collected from the nearest automatic weather stations, where available, or from the Republic Hydrometeorological Service of Serbia.

Assessed localities in Serbia were chosen randomly across the northern Serbian province of Vojvodina and in Belgrade suburbia covering the complete sugar beet production area in Serbia. To obtain representative numbers of ‘*Ca*. P. solani’ strains up to 15 symptomatic sugar beet samples were collected per inspected locality (Table 1). To assess the presence of RTD in sugar beets in Slovakia, where RTD was not previously reported, but RTD symptoms were recently observed on sugar beets by MariboHilleshög ApS (personal communication), 20 RTD-symptomatic and 10 asymptomatic sugar beets were sampled. 

The scattered presence of RTD symptoms on sugar beet was observed during 2020 in Croatia and Hungary by the sugar beet production companies and resulted in collecting 16 and 20 symptomatic plants, respectively. Four sugar beet samples from one location in Austria that were expressing only leaf RTD symptoms were included in the analysis. The targeted 15 samples (20 in the case of Farna, Slovakia) were collected from the four randomly selected rows during estimation of the disease frequency where possible. If due to low disease frequency, the targeted number of plants could not be achieved, additional samples were collected from the symptomatic plants scattered in the fields. All root samples were cross-cut and checked for the presence of discolouration and root rot prior to laboratory analysis. Additionally, one sugar beet sample from Germany, obtained during the SBR survey by a production company, as SBR-symptomatic, but finally resulted ‘*Ca*. A. phytopathogenicus’-negative was also analysed in the presented study (Table 1). 

### 2.2. Nucleic Acid Extraction and ‘Ca. A. phytopathogenicus’ Detection

Nucleic acid extraction from sugar beet and red beet samples was performed from 0.5 g of taproot, following the CTAB protocol [31]. Total nucleic acids were precipitated with isopropanol and resuspended in a TE buffer.

For the ‘*Ca*. A. phytopathogenicus’ detection, PCR using the Fra5/Fra4 primer pair, which allows amplification of the 16Sr RNA gene of ‘*Ca*. A. phytopathogenicus’, was applied to all samples. Conditions for the amplification and reaction mix compositions were the same as described previously [8,32]. Samples lacking template DNA were employed as negative controls, while the ‘*Ca*. A. phytopathogenicus’ strain HN1220/5 from Germany [8] was used as the positive control. The obtained PCR products were separated in 1% agarose gel, stained with ethidium bromide, and visualised with a UV transilluminator.

### 2.3. ‘Ca. P. solani’ Detection and MLSA

The presence of ‘*Ca*. P. solani’ in the collected sugar beet and red beet samples was based on the amplification of the ‘*Ca*. P. solani’-specific *stamp* gene. All *stamp*-positive samples were subjected to sequence analyses and genotyped on *tuf* and *vmp1* genes, resulting in a specific three-loci genotypisation of each ‘*Ca*. P. solani’ strain. Nucleotide sequences of the partial *stamp*, *tuf* and *vmp1* genes from the ‘*Ca*. P. solani’ strains, of each multilocus genotype detected per country in this study, were deposited in GenBank under the accession numbers given in Table 1. In all PCR assays, samples lacking template DNA were employed as negative controls, while the previously characterised ‘*Ca*. P. solani’ strain from sugar beet (429/19) was used as a positive control [8]. Sugar beet samples that were negative in the *stamp* analyses were further subjected to direct PCR assays with the primer pair P1/P7 [33,34] and nested PCR with the primer pair R16F2n/R2 [35] for universal phytoplasma detection. The obtained R16F2n/R2 PCR products were separated and visualised as described above, and commercially sequenced in both directions using the same primers as was used for amplification (Macrogen Inc., Seoul, South Korea).

#### 2.3.1. Stamp Gene

Amplification of the stolbur phytoplasma-specific *stamp* gene was performed in nested PCR assays, using the Stamp-F/R0 and Stamp-F1/R1 primer pairs and following previously described conditions [36]. Each 25 μL PCR mix contained 20 ng of template DNA, 1× PCR Master Mix (Thermo Scientific, Vilnius, Lithuania) and 0.4 μM of each primer. In total, 1 μL of direct PCR amplicon diluted 30× in sterile water was used as a template for the nested PCR. Nested products were separated and visualised as described above. Nested *stamp* amplicons were sequenced, obtained sequences were manually inspected, assembled using Pregap4 from the Staden Package [37] and then aligned with *stamp* genotypes previously described in Central Europe [11,36,38] using ClustalX, under MEGA X [39,40]. The phylogenetic Median-Joining network was constructed in the software NETWORK version 10.2 [41] in order to assess the genetic relatedness of the ‘*Ca*. P. solani’ strains detected in this study, as well as the reference *stamp* strains from central Europe. Settings used in the analysis were keeping the parameter Epsilon at its default value 0 and applying maximum parsimony post-processing to obtain a network containing all the shortest trees.

#### 2.3.2. Tuf Gene

In order to amplify the *tuf* gene, the Tuf1f/r primer pair was followed by fTufAy/rTufAy in nested PCR assays [42]. For those samples in which no, or very weak, amplification was obtained, an alternative semi-nested protocol was used for amplifying the *tuf* gene, applying fusAF1/tufBR1 primers, followed by fusAF2/tufBR1 [16]. Nested amplicons of both PCR systems were separated and visualised as described above. For differentiation of the *tuf* types: tuf-a, b and d, the obtained *tuf* amplicons (fTufAy/rTufAy and fusAF2/tufBR1) were subjected to the RFLP analyses with the *Hpa*II and *Tai*I restriction enzymes (Thermo Scientific) in separate reactions, according to manufacturer’s instructions [8,10]. Restriction products of both RFLP analyses were separated in 8% polyacrylamide gel, stained, and visualised as described above. As reference strains of the three *tuf* types (a, b and d) ‘*Ca*. P. solani’, strains SB1, 284/09 and 429/19 were used, respectively [8,43,44]. All ‘*Ca*. P. solani’ samples determined by the RFLP analysis as tuf-b types, but previously genotyped on the *stamp* gene as members of the cluster tuf-a, were subjected to the sequencing analysis of the *tuf* gene to reveal whether they belong to the tuf-b1 or b2 type [11]. To check for the presence of additional variability in the *tuf* gene, representative ‘*Ca*. P. solani’ strains per each detected multilocus genotype were commercially sequenced on this gene, along with additional 38 strains randomly selected per country and locality, as described above. The sequences of samples amplified with fusAF2/tufBR1 were trimmed to the length of the fTufAy/rTufAy amplicon before multiple sequence alignment, for comparison. Sixty-five samples in total were subjected to *tuf* sequencing.

#### 2.3.3. Vmp1 Gene

To amplify the *vmp1* gene, nested PCR assays were performed using the primer pairs StolH10F1/R1 and TYPH10F/R [45,46]. The obtained nested *vmp1* amplicons were subjected to RFLP analyses with the *Rsa*I restriction enzyme (Thermo Scientific) according to the manufacturer’s instructions. Restriction products were separated and visualised as described above. Samples identified as the V2 profile were subjected to an additional RFLP analysis with *Taq*I and *Alu*I restriction enzymes, separately, to determine whether the samples bear the V2-TA type [47]. Nested PCR products of the three samples from Serbia that had a unique *vmp1* profile previously not described, and representative ‘*Ca*. P. solani’ strains per each detected multilocus genotype in all assessed countries, were sequenced. The ‘*Ca*. P. solani’ strain from Germany was subjected only to sequencing. All obtained *vmp1* sequences were aligned and compared with those of ‘*Ca*. P. solani’ strains representing previously described *vmp1* RFLP profiles, as described above [48,49]. A virtual RFLP gel was generated using the software pDRAW32 (http://www.acaclone.com/ (accessed on 15 April 2021)). The evolutionary history was inferred based on the partial *vmp1* gene sequences of ‘*Ca*. P. solani’ strains representing each of the *vmp1* profiles detected in this study and reference strains of previously described profiles [48,49], using the Maximum Likelihood (ML) method (MEGA X). A sequence evolution model was chosen using the “find best model” option in MEGA X. Initial tree(s) for the heuristic search were obtained automatically by applying the Neighbor-Join and BioNJ algorithms to a matrix of pairwise distances estimated using the Maximum Composite Likelihood approach and then selecting the topology with superior log likelihood value. To estimate the statistical significance of the inferred clades, 1000 bootstraps were performed. 

## 3. Results

### 3.1. RTD, with Its Associated Agent ‘Ca. P. solani’, Is Present in All Assessed Countries in the Pannonian Plain

During the survey, the typical RTD symptoms (i.e., yellowing and necrosis of leaves and wilting and rubberiness of roots) were observed on sugar beet at all assessed locations in Serbia and in Farna, Slovakia. Neither the typical SBR symptom (discolouration of root vascular tissue) nor symptoms and signs of root pathogens (root rot and any other symptom) were observed. At two locations in Serbia (Rimski Šančevi and Bačko Dobro Polje), symptomatic plants with milder root symptoms were observed, i.e., the tail of the taproot was flexible, while the root crown was less rubbery. These two sites were the only ones that had significantly higher (more than double) than normal levels of precipitation in August 2020 of 138 mm and 106 mm, respectively (1981–2015 average is 55 mm). Typical RTD symptoms were observed in both analysed localities in Hungary, as well as in Novi Jankovci (CRO), while in Beli Manastir (CRO) only one plant expressing the symptoms was found and collected.

According to disease incidence, the assessed sugar beet fields were separated into two groups—(1) localities with non-epidemic RTD occurrence, with low disease frequency and symptomatic plants scattered across the fields and (2) localities with epidemic RTD occurrence, with high disease frequency and numerous symptomatic plants. In 8 out of the 11 inspected sugar beet localities in Serbia, due to very low disease incidence, areas with the highest numbers of symptomatic plants, for setting the estimation plot, could not even be visually determined. Therefore, the low disease frequency (≤0.5%) was estimated only based on four randomly selected 60 m-long rows, classifying those localities as non-epidemic. On the other hand, in the other three sugar beet localities in Serbia, and the one in Slovakia, the quadrant with the highest disease frequency was easily determined, and high RTD incidence was confirmed according to both methods, the randomly selected rows and the estimation plot. Those four localities were considered as the ones with epidemic disease occurrence. Non-epidemic RTD occurrence was observed at the red beet locality in Stepanovićevo (SRB). From each of the three localities with high RTD incidence in Serbia, 15 sugar beets were sampled, while a total of 111 sugar beets were collected from the eight sites with low disease frequency. As a result, a total of 156 sugar beet samples and 12 red beet samples from Serbia were further analysed. In Farna, Slovakia, where high disease frequency was observed, 20 RTD-symptomatic and 10 asymptomatic sugar beet samples were collected since there is no previous data of the RTD occurrence in this country. In Hungary and Croatia low disease incidence was found at all four assessed sites. Four samples from Austria that were included in the analysis did not express the typical RTD root symptom, i.e., a rubbery taproot, but had prominent leaf yellowing and necrosis with secondary leaf growth emerging laterally from the root neck among the collapsed necrotic leaves. Overall, 216 RTD-symptomatic sugar beet samples from the Pannonian Plain, one non-SBR sugar beet sample from Germany and 12 red beets from Serbia were analysed in the presented study (Table 1).

The presence of ‘*Ca*. P. solani’ was revealed in sugar beet samples from all localities assessed in five assessed countries of the Pannonian Plain (Serbia, Slovakia, Croatia, Hungary and Austria) and red beets from Serbia. A total of 205 out of 216 (95%) RTD-symptomatic sugar beets, and 10 out of 12 symptomatic red beets, were infected with stolbur phytoplasma (Table 1). In Farna, Slovakia, all 20 RTD-symptomatic samples were infected with ‘*Ca*. P. solani’, while all 10 asymptomatic ones were negative. All 16 sugar beet samples from the two locations in Croatia were infected with ‘*Ca*. P. solani’, as were 20 out of the 21 samples from the two locations in Hungary. Four sugar beets obtained from Rutzendorf, Austria without typical RTD root symptoms were ‘*Ca*. P. solani’-positive. One sample from Germany, additional included in the analysis, was also infected with stolbur phytoplasma. The main organism associated with the SBR, ‘*Ca*. A. phytopathogenicus’, was not detected in any of 216 analysed sugar beet samples from the Pannonian Plain, the one in Germany nor in 12 red beet samples. Using the universal phytoplasma system on two sugar beets, namely one from Serbia (Bačko Dobro Polje) and one from Hungary (Tamási), ‘*Ca*. P. asteris’ (16SrI) was detected, while in two other samples from Serbia (Stari Tamiš) presence of ‘*Ca*. P. prunorum’ (16SrX) was revealed. The four detected strains of ‘*Ca*. P. asteris’ and ‘*Ca*. P. prunorum’ were excluded from further molecular characterisation.

### 3.2. Multilocus Characterization of the RTD-Associated ‘Ca. P. solani’ Strains Revealed a High Overall Diversity, but a Low Diversity at Localities with Epidemic Disease Occurrence

#### 3.2.1. Stamp Genotypisation Showed Predominance of Genotypes Related to tuf-b1/tuf-d Types and Minor Presence of the Ones Related to tuf-a/tuf-b2 in Sugar Beet

All obtained ‘*Ca*. P. solani’ strains were molecularly characterised on the *stamp* gene, resulting in the detection of 14 distinct *stamp* genotypes in sugar beet and three in red beet (Table 1). Seven out of fourteen genotypes from the sugar beet were previously found in Serbia (STOL, Rpm35, M5, Rqg31, and Rqg50) and central Europe (GGY and SB5), while another seven *stamp* genotypes are detected for the first time in this study: RTD1, RTD2, RTD3, RTD4, RTD5, RTD6, and Z187 (Table 1). Genotypes RTD1–5 were found only in Serbia, each detected per single sample. A new *stamp* genotype, RTD6, present in one sugar beet in Sombor, Serbia, was also detected in three samples at Tamási, Hungary. Genotype Z187 was found only in the one sugar beet sample from Germany (Table 1).

The prevailing *stamp* genotype was STOL, present at all localities. It was found in 120 out of 145 ‘*Ca*. P. solani’-positive samples from Serbia, and in as many as 173 out of a total of 205 ‘*Ca*. P. solani’-positive sugar beet samples from across the Pannonian Plain (Table 1, Figure 1). Other *stamp* genotypes had a considerably minor presence. Genotype Rqg31 was present in Serbia at six localities, GGY and Rqg50 at two, M5 at one, as well as the genotype SB5. Genotype GGY was detected in two samples from Austria and one from Hungary where Rqg31 was also present. A total of three *stamp* genotypes were detected in the red beet samples: Rqg31, Rqg50 and M5 (Table 1).

The median joining phylogenetic network of the *stamp* gene confirmed the presence of the four genetic clusters within which the newly described genotypes are incorporated (Figure 1). In cluster tuf-a, comprising *stamp* genotypes associated with tuf-a and b2 types, the previously known SB5 genotype is by nine nucleotide differences delineated from the diversifying group of strains that includes two newly detected *stamp* genotypes, RTD2 (SRB) and RTD6 (SRB, HUN), both with a single nucleotide difference from the closest strains, 19–25 and StAt2, respectively. Genotypes associated with the tuf-b1 and tuf-d types form three clusters in the network. The new genotype Z187, detected in the sample from Germany, is associated with the *stamp* genotypes LG, PO, StolC and H299 from France. Six SNPs connect this group to the neighbouring cluster I tuf-b. The most commonly found genotype in the analysed sugar beet samples, STOL, along with the genotypes Rpm35 and M5, form this cluster. Two new genotypes, RTD1 and RTD5 (SRB), are closely related to the STOL genotype and clustered within this genetic group. Cluster III tuf-b is formed by a diverse group of *stamp* genotypes, Rqg31 (SRB), Rqg50 (SRB) and GGY (SRB, AUT), and supplemented by the two new genotypes, RTD3 and RTD4, both found in Serbia.

#### 3.2.2. Tuf Genotypisation Revealed Presence of All Four tuf Types Previously Known in Europe, with Prevalence of the tuf-d Type

Using the fTufAy/rTufAy primer pair in the nested PCR protocol, expected length fragment amplification was obtained in 115 out of 205 ‘*Ca*. P. solani’ positive sugar beet samples from the five countries in the Pannonian plain and in one sample from Germany, but in none of the 10 positive red beets. Of the remaining 90 sugar beet samples, amplification of the *tuf* gene was successfully performed in 83 samples using the alternative protocol, (fusAF2/tufBR1 in the nested PCR) as for all 10 red beets. Finally, in a total of seven sugar beet samples, *tuf* gene amplification was not successful with any of the used protocols. Results of the RFLP analyses revealed three *tuf* types among strains from the sugar beet. The most common was tuf-d, followed by tuf-b, while tuf-a was detected only in one sample (Table 1). Sequencing of the four tuf-b strains, whose *stamp* genotypes corresponded to the cluster tuf-a, revealed the presence of the tuf-b2 type, as expected. In red beet, only tuf-b1 was detected. Overall, the tuf-d type was detected in 148 sugar beet samples (74.4%), b1 in 46 (23.1%), while tuf-a and tuf-b2 were present in one (0.5%) and four (2%) samples, respectively. Multiple sequence alignment of 65 strains showed no intra-tuf type variability (data not shown). Sequence analysis of the ‘*Ca*. P. solani’ strain Z187 from Germany showed its tuf-b1 affiliation according to the *tuf* type-specific restriction sites but revealed an SNP difference on position 507 after the *tuf* start codon (T/C, which is synonymous [Pro] when translated) when compared to other strains belonging to the tuf-b1 type.

#### 3.2.3. Vmp1 RFLP Analyses Revealed Five Previously Described and One New Profile

**Figure 2:**
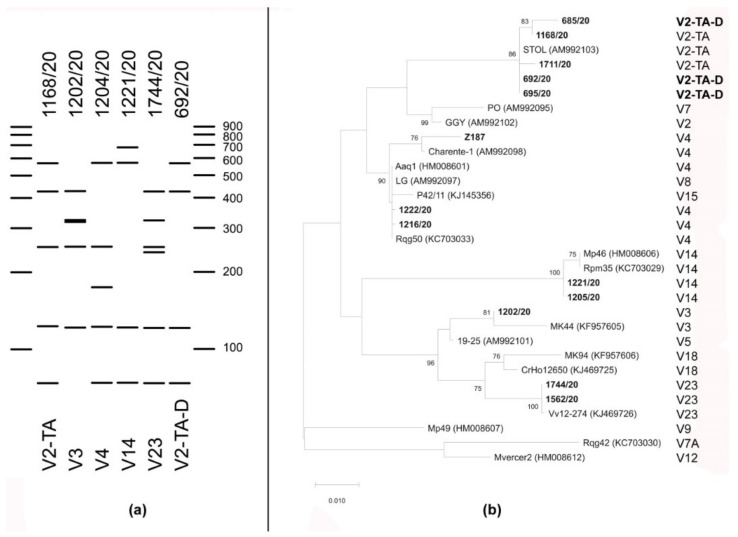
Analyses of the *vmp1* gene (TYPH10F/R amplicon). (**a**) Representative virtual RFLP gel showing the *vmp1* patterns digested with the *Rsa*I restriction enzyme of the ‘*Ca*. P. solani’ strains obtained in this work (stated above) with corresponding *vmp1* profile (stated below) of the reference ‘*Ca*. P. solani’ isolate shown in panel b. Fragment sizes of the marker are indicated in base pairs. (**b**) The evolutionary history was inferred using the Maximum Likelihood method applying the GTR model of 31 *vmp1* sequences of ‘*Ca*. P. solani’ strains obtained in this work and selected reference strains. The tree with the highest log-likelihood is shown. Accession numbers are given in parentheses. The tree is drawn to scale, with branch lengths measured in numbers of substitutions per site. *Vmp1* sequences of the ‘*Ca*. P. solani’ strains obtained in this work and newly described restriction patterns are in bold.

Amplification of the partial *vmp1* gene was achieved in 194 of 205 ‘*Ca*. P. solani’-infected sugar beet and in all 10 red beet samples. RFLP analyses showed that six different RFLP profiles could be distinguished among the analysed samples (Table 1). Five of the obtained profiles (V2-TA, V3, V4, V14 and V23) corresponded to those already described in the literature (Figure 2) [48,49], while three samples from Erdevik (SRB) had a unique new profile that could not be assigned to any of those previously described (Table 1). Virtual RFLP analyses confirmed the assignment of the analysed strains to previously described ones and the presence of the new *vmp1* profile entitled V2-TA-D (Figure 2a). Comparing the *vmp1* sequence of the new RFLP profile with reference sequences of the V2-TA type (strain STOL) showed a 252nt deletion in strains from Erdevik, that encompasses one restriction site resulting in the absence of the 252 bp restriction fragment in the RFLP pattern of the new profile. Another three restriction sites characteristic of the V2-TA profile are present in the sequences of both *vmp1* profiles. Therefore, the suffix “D” was appended to the already known acronym V2-TA, to indicate the specific deletion described above. Virtual RFLP analyses for strain Z187 from Germany showed that it bears the *vmp1* profile V4, while sequence analysis showed the presence of the same restriction sites as in the V4 profile (strains Charente-1 and Rqg50), albeit with a six nt-deletion. Instead of two fragments of the same size of 252 bp present in V4 profile, Z187 has two fragments of unequal length, 252 bp and 246 bp, although this does not visibly alter the pattern of the V4 RFLP profile (Figure 2a).

The General Time Reversible (GTR) model of nucleotide substitution was applied for the phylogenetic analysis of the partial *vmp1* sequences. A total of 13 ‘*Ca*. P. solani’ strains from sugar beet representing the *vmp1* RFLP profiles obtained in this work and 18 representative sequences of previously described profiles were included in the analysis (Figure 2a). The unrooted *vmp1* phylogenetic tree indicated clustering of the representative ‘*Ca*. P. solani’ strains from sugar beet with corresponding reference strains of the same RFLP profile. No separate genetic cluster was formed for the strains 685/20, 692/20, and 695/20, bearing newly described *vmp1* profile V2-TA-D, but they clustered with strains of the reference V2-TA profile (strain STOL). Moreover, the reference strain of the V2 profile (strain GGY) did not cluster with the V2-TA strains but is shown to be closer to the reference strain of V7 (strain PO). Strain Z187 from Germany, bearing the V4 profile with the six nt-deletion, clusters with other strain of the V4 type identified in this study and the reference strains of the same profile (strains Charente-1, Aaq1, LG, P42/11, Rqg50).

#### 3.2.4. MLSA Revealed Prevalence of the Newly Described Three Loci Genotype dSTOLg with Its Exclusive Presence on Six Localities

**Figure 3:**
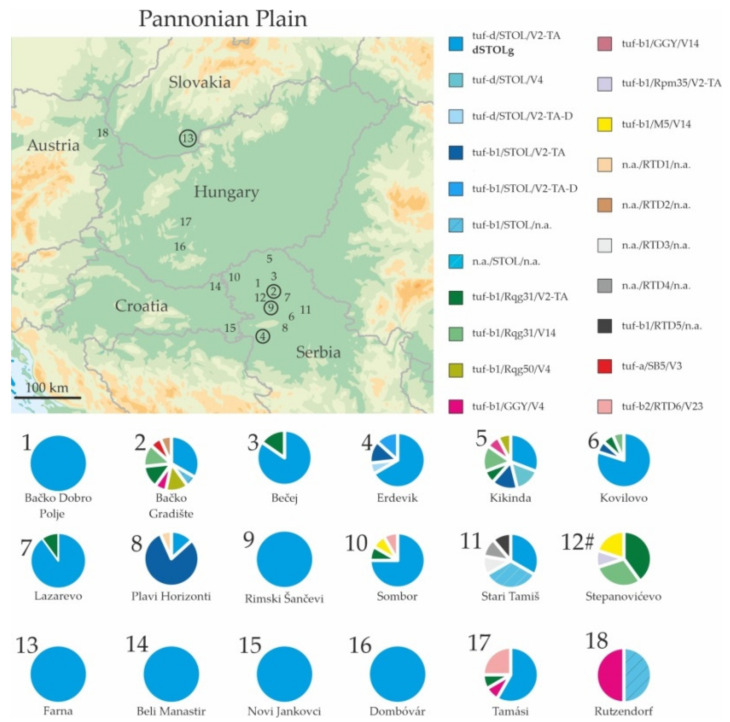
Map of the Pannonian Plain depicting sampling localities of the RTD-symptomatic sugar beets and red beets. Numbers on the map correspond to the collecting sites given in Table 1. Encircled numbers denote localities with epidemic RTD occurrence. Chart sections represent the proportion of the specific ‘*Ca*. P. solani’ multilocus genotypes following the specified colour pattern given in the legend on the right. A total of 21 comprehensive genotypes detected in this study are presented by different colours. [11,12,38,47,50,51]. Unamplified genes within comprehensive genotypes are marked with the acronym “n.a.”.

The complete, three loci-genotypisation of the ‘*Ca*. P. solani’ strains from sugar beets was successful in 194 out of 205 samples from the Pannonian plain and from the one from Germany. Characterisation of the assessed genes (*tuf*/*stamp*/*vmp1*) resulted in detecting 14 (five new) distinct three-gene genotypes in the Pannonian Plain (Table 1, Figure 3). Incomplete genotypisation was obtained in 11 ‘*Ca*. P. solani’ samples resulting in seven partially characterised genotypes, two of them associated with *stamp* STOL strains and five with the newly described *stamp* genotypes RTD1, 2, 3, 4, and 5.

The prevailing multilocus genotype in sugar beet, present in 145 samples (70.4%), was tuf-d/STOL/V2-TA, designated dSTOLg (Table 1, Figure 3). It was found at all localities in Serbia, being the only genotype present at two sites with epidemic and non-epidemic RTD occurrence (Rimski Šančevi and Bačko Dobro Polje, respectively). Genotype dSTOLg was the single genotype found in Farna, Slovakia and at the two localities in Croatia, while in Hungary it was detected in all samples from Dombóvár, and seven out of 12 sugar beets in Tamási (Table 1). Only two tuf-d/STOL strains were affiliated to the V4 *vmp1* profile (Kikinda/SRB). Merely 17 sugar beet samples, all from Serbia, had the tuf-b1/STOL/V2-TA (STOLg) multilocus genotype. Most genetic variants associated with the *stamp* STOL genotype were detected at locality Erdevik (SRB), with epidemic RTD occurrence, including the new *vmp1* profile, V2-TA-D, occurring with both, tuf-d/STOL and tuf-b1/STOL strains. Additionally, the STOL *stamp* genotype associated with tuf-b1 was detected at Rutzendorf (AUT).

Unlike the tuf-d type, linked exclusively to the STOL *stamp* genotype, the b1 type was in Serbia present within seven other comprehensive genotypes: tuf-b1/Rqg31/V2-TA, tuf-b1/Rqg31/V14, tuf-b1/Rqg50/V4, tuf-b1/GGY/V4 tuf-b1/GGY/V14 tuf-b1/Rpm35/V2-TA and tuf-b1/M5/V14 [11,12,47,50] (Table 1 and Table S1, Figure 3). Multilocus genotypes of the tuf-b1 type detected in Hungary were tuf-b1/Rqg31/V2-TA and tuf-b1/GGY/V14 (Tamási), while in Rutzendorf (AUT) tuf-b1/GGY/V14 was present. In Germany, the only ‘*Ca*. P. solani’ multilocus genotype present, i.e., tuf-b1/Z187/V4, was not found at any of the assessed localities in the Pannonian Plain, nor was reported previously. A minor occurrence of the ‘*Ca*. P. solani’ genotypes affiliated to the tuf-a and b2 types was detected in Serbia as comprehensive genotype tuf-a/SB5/V3 [38] and newly described tuf-b2/RTD6/V23. The latter was detected also in Hungary (Tamási) (Table 1, Figure 3).

All 10 ‘*Ca*. P. solani’ strains from the red beet were genotyped on all three loci. The prevalent were genotypes tuf-b1/Rqg31/V2-TA and tuf-b1/Rqg31/V14 (seven samples). Two samples had the multilocus genotype tuf-b1/M5/V14, and one tuf-b1/Rpm35/V2-TA.

## 4. Discussion

The results of the present study confirm the association of sugar beet RTD with ‘*Ca*. P. solani’ presence across the Pannonian Plain and extends previous data on RTD occurrence and associated ‘*Ca*. P. solani’ characterisation gathered in Serbia [8]. However, RTD occurrence greatly varied across the assessed localities, corresponding to previous reports on spatial and temporal variations in disease dynamics with epidemic and non-epidemic periods [8,52]. A similar finding was documented for ‘*Ca*. P. solani’-associated diseases of other annual crops (e.g., corn reddening, pepper yellow wilt) [53,54]. RTD occurrence was revealed throughout sugar beet-growing areas in Serbia, and for the first time, it was detected in Slovakia, where the disease outbreak was observed in 2020 and perceived as a limiting factor in terms of production (MariboHilleshög ApS, Holeby, Denmark, personal communication). Moreover, at Stepanovićevo in Serbia, RTD-like symptoms and the associated agent ‘*Ca*. P. solani’ were detected in red beet–unsurprisingly, as both red and sugar beet belong to the same subspecies, namely *Beta vulgaris* subsp. *vulgaris*. Milder root rubberiness was observed on several of the collected samples at Rimski Šančevi and Bačko Dobro Polje in Serbia. These sites had precipitation significantly above average during the period of intensive RTD symptom development (August), thereby suggesting that the development of the root rubberiness, an RTD symptom of particular economic interest due to its impact on further processing, could be dependent on soil moisture. This environmental factor could affect the severity of root symptom expression, but not disease frequency. Previous data on the sugar beet RTD in Serbia (then of unknown aetilogy) report more severe disease occurrence in dry years, supporting this study [52]. The absence of root symptoms in the four ‘*Ca*. P. solani’-infected sugar beets from the location in Austria could also be due to the higher soil moisture. Moreover, a lack of root rubberiness in the ‘*Ca*. P. solani’-infected sugar beets in France could likewise be correlated to variations in soil moisture, while the typical SBR symptom, i.e., vascular root tissue discolouration, remained [24]. SBR-associated ‘*Ca*. A. phytopathogenicus’ was not detected in any of the 156 sugar beets, or the 12 red beets collected in Serbia, the same applied to the other assessed countries of the Pannonian Plain. This indicates that this prokaryote is not involved in RTD aetiology in Serbia, since the sugar beet samples were comprehensively collected throughout all production areas, which makes it unlikely that ‘*Ca*. A. phytopathogenicus’ remained undetected. Although RTD and SBR symptoms are clearly distinguishable by a brownish vascular discolouration present in the root cross-section in the case of SBR, but not in the case of RTD, they could be overlooked during routine sampling by a production company. This is the first report of ‘*Ca*. P. solani’ in sugar beet in Germany, and therefore the local presence of SBR and its associated agent ‘*Ca*. A. phytopathogenicus’ [22], should not distract further research from recognising the importance of phytoplasmas in sugar beet diseases in Germany.

‘*Ca*. P. solani’ in Central Europe has been associated with at least two epidemiological cycles, namely *H. obsoletus*-*C. arvensis* and *H. obsoletus*-*U. dioica*, which are traceable in the specific agroecosystem through the genetic variability of the three loci analysed in this work [10,11]. Accordingly, the overall high genetic variability, particularly in the case of *tuf*, an epidemiologically informative marker gene, of the ‘*Ca*. P. solani’ strains genotyped in this study suggests the presence of more than one ecological cycle in RTD epidemiology. The prevalence of the dSTOLg (tuf-d/STOL/V2-TA) multilocus genotype in RTD-affected sugar beet is the most prominent MLSA finding. In the phylogenetic analyses of the *vmp1* gene, clustering of the ‘*Ca*. P. solani’ strains is congruent with previous studies [48].

The comprehensive genotype STOLg (tuf-b1/STOL/V2-TA) prevails the overall dominant dSTOLg only in one locality in Serbia (Plavi Horizonti). Previously STOLg has been associated with specific ‘*Ca*. P. solani’ epidemiology guided by *H. obsoletus ex C. foetida* in southeast Serbia and another vector, *Reptalus panzeri*, in epidemiology of Bois noir on grapevine [13,47]. Multilocus genotypes tuf-b1/Rqg31/V2-TA, tuf-b1/Rqg31/V4 and tuf-b1/Rqg50/V4 have been previously linked to other *H. obsoletus* association, *H. obsoletus ex C. arvensis*, while the multilocus genotype tuf-b1/M5/V14 has been reported as involved in potato stolbur disease in Serbia [13,50]. Only five ‘*Ca*. P. solani’ strains bearing *tuf* types a and b2, and/or *stamp* genotypes SB5, RTD2, and RTD6 suggest a minor, even negligible, role of the *H. obsoletus*-*U. dioica* pathosystems in the RTD epidemiology. It is corroborated with the results of the previous experiments in which the highly infected *H. obsoletus ex U. dioca* population from France failed to transmit ‘*Ca*. P. solani’ to sugar beet [24].

The limited genetic variability of outbreak strains during epidemics, sometimes with the predominance of a single genotype, is known for pathogens of both plants and animals, including vector-borne ones [55,56,57,58,59]. Accordingly, the majority of ‘*Ca*. P. solani’ genetic variability was detected at localities with non-epidemic disease occurrence, while prominent multilocus genotype dSTOLg was present as the only or prevailing strain in sugar beet fields with epidemics, both in Serbia and Slovakia. This ‘*Ca*. P. solani’ genotype was prevalent in several non-epidemic sites in Serbia and all four assessed localities in Croatia and Hungary, thus pointing to the epidemiological relevance of dSTOLg in RTD outbreaks. The predominant genotype dSTOLg was shown to be responsible for the RTD outbreaks at locations with epidemics in Serbia and Slovakia, despite their spatial distance (situated on the opposite sides of the Pannonian Plain) and the high overall diversity of the ‘*Ca*. P. solani’ strains. Since sugar beet is an annual plant, it represents a dead-end host, most probably infected by an insect vector’s erratic feeding. This may suggest that a specific epidemiological cycle could be responsible for RTD outbreaks, overwhelming other pathways of the ‘*Ca*. P. solani’ dissemination, or even other phytoplasmas that are present during the non-epidemic phase, possibly circulating in the field within their ecological cycles. It is of the utmost importance to assess epidemiology when and where the pathogen is present in the epidemic phase. Accordingly, the pronouncement of the epidemiological cycle(s) traceable during the non-epidemic phases as dominant pathways responsible for disease outbreaks may be misleading. A combination of various ecological and anthropogenic factors could influence the complex relationship among the pathogen, vector(s), host plants and the annual crop-usually a dead-end host, and trigger the epidemics. It raises concern if such a chain of events leading to RTD epidemics with greater severity may be possible on a wider area than previously recorded across the Pannonian Plain. The ‘*Ca*. P. solani’ strain detected in Germany is unique on all three assessed loci compared to the strains from the Pannonian Plain, as well as others previously described. Since the only available genotypisation of the ‘*Ca*. P. solani’ infecting sugar beet from west Europe (France) was based on RFLP analyses of *tuf* gene, it is not possible to compare genetic characteristics of these geographically related ‘*Ca*. P. solani’ strains.

The presence of phytoplasmas, other than ‘*Ca*. P. solani’, in sugar beets (‘*Ca*. P. asteris’ and ‘*Ca*. P. prunorum’, per two samples each) is probably due to the erroneous feeding of insect vectors since these phytoplasmas are present in Serbia on different hosts [60,61]. ‘*Ca*. P. asteris’, a phytoplasma with an extremely wide host plant range, has been reported in several polyphagous insect vector species, while for ‘*Ca*. P. prunorum’ there are reports of the infection of non-*Prunus* plant species [62,63,64]. Although, the presence of phytoplasmas other than *‘Ca*. P. solani’ in sugar beet is most probably irrelevant in the context of the RTD outbreaks, it is still noteworthy to mention that this finding is an example of “spillover” and how a host shift may occur [38,65]. Sugar beets infected with non-’*Ca*. P. solani’ phytoplasmas have expressed typical RTD symptoms. This finding, supplemented with data on the 16SrIII-J phytoplasmas occurrence on sugar beet in South America, supports consideration of the association of phytoplasmas with other unelucidated sugar beet diseases with similar symptomatology such as the “rubber root” in Arizona, USA [66,67,68].

## 5. Conclusions

The results of the conducted survey highlight the presence of sugar beet RTD in all assessed countries across the Pannonian Plain. The survey represents a comprehensive study of RTD in Serbia, but an initial study in other countries of the Pannonian Plain and Germany. Moreover, the results reveal the prevalence of the single multilocus genotype, dSTOLg, in almost all localities, relating this ‘*Ca*. P. solani’ strain to the epidemic RTD occurrence in Serbia and Farna, Slovakia. Thorough molecular characterisation of the ‘*Ca*. P. solani’ strains infecting sugar beet, presented in this study, is essential for setting up a future hypothesis on the RTD epidemiology. Pursuing weeds and potential insect vectors for the ‘*Ca*. P. solani’ multilocus genotypes detected in sugar beets in this study, will be a prime goal in the following years, focusing on the pathways of the dSTOLg genotypes when assessing the epidemiological situation in the field.

## Figures and Tables

**Table 1:** RTD-symptomatic sugar beet and red beet samples analysed in this work, as well as obtained ‘*Ca*. P. solani’ multilocus genotypes.

Locality/Country (GPS)	RTD Incidence	Phytoplasma Positive/Tested Samples	*tuf*/*stamp*/*vmp1* Genotype	No. of Samples	Representative Strain	Acc. No. *tuf/stamp*/*vmp1*
1. Bačko Dobro Polje/SRB (45°31′59.7″ N 19°40′49.2″ E)	0.1%	15/15^1^	tuf-d/STOL/V2-TA	14		
**2. Bačko Gradište/SRB** (45°31′03.7″ N 19°59′25.1″ E)	**5.7%**	15/15	tuf-d/STOL/V2-TA	5		
			tuf-b1/STOL/n.a.	1		
			tuf-b1/Rqg50/V4	2	1204/20	MZ927444/MZ604949/OK041397
			tuf-b1/GGY/V14	1	1205/20	MZ927445/MZ604950/OK041401
			tuf-b1/Rqg31/V2-TA	2		
			tuf-b1/Rqg31/V14	2		
			tuf-a/SB5/V3	1	1202/20	MZ927432/MZ604951/OK041396
			n.a./**RTD2**/n.a.	1	1198/20	n.a./MZ604952/n.a.
3. Bečej/SRB (45°39′56.2″ N 19°53′19.0″ E)	0.1%	13/13	tuf-d/STOL/V2-TA	11		
			tuf-b1/Rqg31/V2-TA	2	1711/20	MZ927454/MZ604959/OK041389
**4. Erdevik/SRB** (45°06′20.1″ N 19°24′31.1″ E)	**7.2%**	15/15	tuf-d/STOL/V2-TA	10		
			tuf-d/STOL/**V2-TA-D**	1	685/20	MZ927429/MZ604944/OK041385
			tuf-b1/STOL/V2-TA	2	696/20	MZ927430/MZ604945/OK041387
			tuf-b1/STOL/**V2-TA-D**	2	692/20	MZ927431/MZ604946/OK041386
5. Kikinda/SRB (45°50′58.1″ N 20°25′10.2″ E)	0.4%	13/14	tuf-d/STOL/V2-TA	4		
			tuf-d/STOL/V4	2	1222/20	MZ927449/MZ604953/OK041399
			tuf-b1/STOL/V2-TA	2		
			tuf-b1/Rqg31/V2-TA	1		
			tuf-b1/Rqg31/V14	2	1221/20	MZ927447/MZ604954/OK041402
			tuf-b1/GGY/V4	1	1216/20	MZ927446/MZ604955/OK041398
			tuf-b1/Rqg50/V4	1		
6. Kovilovo/SRB (44°53′30.8″ N 20°24′58.8″ E)	0.4%	15/15	tuf-d/STOL/V2-TA	12		
			tuf-b1/STOL/V2-TA	1		
			tuf-b1/Rqg31/V2-TA	1		
			tuf-b1/Rqg31/V14	1		
7. Lazarevo/SRB (45°25′14.8″ N 20°31′21.2″ E)	0.1%	10/12	tuf-d/STOL/V2-TA	9	1168/20	MZ927433/MZ604947/OK041388
			tuf-b1/Rqg31/V2-TA	1		
8. Plavi Horizonti/SRB (44°52′08.4″ N 20°15′58.7″ E)	0.4%	15/15	tuf-d/STOL/V2-TA	2		
			tuf-b1/STOL/V2-TA	12		
			n.a./**RTD1**/n.a.	1	1177/20	n.a./MZ604948/n.a.
**9. Rimski Šančevi/SRB** (45°19′47.0″ N 19°49′10.6″ E)	**10.3%**	15/15	tuf-d/STOL/V2-TA	15		
10. Sombor/SRB (45°47′19.0″ N 19°04′33.0″ E)	0.3%	12/12	tuf-d/STOL/V2-TA	9		
			tuf-b1/Rqg31/V2-TA	1		
			tuf-b1/M5/V14	1	1746/20	MZ927453/MZ604960/OK041407
			tuf-b2/**RTD6**/V23	1	1744/20	MZ927434/MZ604961/OK041409
11. Stari Tamiš/SRB (44°52′31.3″ N 20°45′42.6″ E)	0.1%	11/15 ^2^	tuf-d/STOL/V2-TA	3		
			n.a./STOL/n.a.	3		
			n.a./**RTD3**/n.a.	1	1443/20	n.a./MZ604956/n.a.
			n.a./**RTD4**/n.a.	1	1447/20	n.a./MZ604957/n.a.
			tuf-b1/**RTD5**/n.a.	1	1453/20	MZ927435/MZ604958/n.a.
12. # Stepanovićevo/SRB (45°25′22.5″ N 19°40′47.2″ E)	0.3%	10/12	tuf-b1/Rqg31/V2-TA	4	1432/20	MZ927448/MZ604962/OK041390
			tuf-b1/Rqg31/V14	3	1438/20	MZ927450/MZ604963/OK041404
			tuf-b1/Rpm35/V2-TA	1	1435/20	MZ927436/MZ604964/OK041391
			tuf-b1/M5/V14	2	1429/20	MZ927437/MZ604965/OK041403
**13. Farna/SVK** (48°01′18.8″ N 18°31′36.2″ E)	**9.5%**	20/20	tuf-d/STOL/V2-TA	20	1589/20	MZ927438/MZ604966/OK041395
14. Beli Manastir/CRO (45°49′38.9″ N 18°37′02.2″ E)	0%	1/1	tuf-d/STOL/V2-TA	1		
15. Novi Jankovci/CRO (45°15′03.0″ N 18°52′39.0″ E)	0.2%	15/15	tuf-d/STOL/V2-TA	15	1512/20	MZ927439/MZ604967/OK041392
16. Dombóvár/HUN (46°23′36.0″ N 18°08′44.3″ E)	0.1%	8/8	tuf-d/STOL/V2-TA	8	1572/20	MZ927452/MZ604971/OK041394
17. Tamási/HUN (46°41′07.5″ N 18°17′34.5″ E)	0.1%	13/13 ^1^	tuf-d/STOL/V2-TA	7		
			tuf-b1/GGY/V14	1	1547/20	MZ927440/MZ604968/OK041405
			tuf-b1/Rqg31/V2-TA	1	1566/20	MZ927451/MZ604969/OK041393
			tuf-b2/**RTD6**/V23	3	1562/20	MZ927441/MZ604970/OK041408
18. Rutzendorf/AUT (48°12′40.4″ N 16°37′42.9″ E)	0%	4/4	tuf-b1/GGY/V14	2	1595/20	MZ927442/MZ604972/OK041406
			tuf-b1/STOL/n.a.	2	1597/20	MZ927443/MZ604973/n.a.
19. Bickenbach/DEU	/	1/1	tuf-b1/**Z187**/V4	1	Z187	MZ927455/MZ604974/OK041400

Localities and RTD incidences with epidemic RTD occurrence, newly described *stamp* genotypes and *vmp1* profiles are given in bold; # red beet locality; ^1^ in one sample ‘*Ca*. P. asteris’ was detected, ^2^ in two samples ‘*Ca*. P. prunorum’ was detected; n.a. not amplified.

## Data Availability

DNA sequences of the assessed genes are available in the GenBank database, accession numbers are given in the Table 1. The Materials and Methods section. All other relevant data are within the paper.

## Supplementary Materials

The following are available online at https://www.mdpi.com/article/10.3390/microorganisms9091950/s1, Table S1: Multilocus genotypisation of RTD associated ‘*Ca*. P. solani’ from the sugar beet.
