# Supplementary material for: Multilocus Genotyping of ‘Candidatus Phytoplasma solani’ Associated with Rubbery Taproot Disease of Sugar Beet in the Pannonian Plain
Source: Microorganisms. 2021 Sep 14;9(9):1950. doi: 10.3390/microorganisms9091950 (PMC8470437; doi:10.3390/microorganisms9091950)
Supplement: Supplementary file 1 [file microorganisms-09-01950-s001.zip › microorganisms-1354492-supplementary.pdf]

Table S1. Multilocus genotypisation of RTD associated '*Ca*. P. solani' from the sugar beet.

Comprehensive genotype[1]	country	No. of samples	Previously assigned genotype[2]	reference
tuf-d/STOL/V2-TA *dSTOLg*	Serbia	94	/	This work
	Slovakia	20		
	Hungary	15		
	Croatia	16		
tuf-d/STOL/V4	Serbia	2	/	This work
tuf-d/STOL/V2-TA-D	Serbia	1	/	This work
tuf-b1/STOL/V2-TA	Serbia	17	STOLg	[43]
tuf-b1/STOL/V2-TA-D	Serbia	2	/	This work
tuf-b1/Rqg31/V2-TA	Serbia	8	Rqg31=CPsM4_AT14	[9, 43]
	Hungary	1		
tuf-b1/Rqg31/V14	Serbia	5	tuf-b1/Rqg31/V14	[43]
tuf-b1/Rqg50/V4	Serbia	3	Rqg50=CPsM4_At12	[9, 43]
tuf-b1/GGY/V4	Serbia	1	CPsM4_At9	[43]
tuf-b1/GGY/V14	Serbia	1	CPsM4_At7	[43]
	Hungary	1		
	Austria	2		
tuf-b1/M5/V14	Serbia	1	tuf-b1/M5/V14	[46]
tuf-a/SB5/V3	Serbia	1	tuf-a/S5/N12	[34]
tuf-b2/RTD6/V23	Serbia	1	/	This work
	Hungary	3		
tuf-b1/Z187/V4	Germany	1	/	This work
tuf-b1/STOL/n.a.	Serbia	1	/	This work
	Austria	2		
n.a./STOL/n.a.	Serbia	3	/	This work
n.a./RTD1/n.a.	Serbia	1	/	This work
n.a./RTD2/n.a.	Serbia	1	/	This work
n.a./RTD3/n.a.	Serbia	1	/	This work
n.a./RTD4/n.a.	Serbia	1	/	This work
n.a./RTD5/n.a.	Serbia	1	/	This work

[1]n.a. not amplified; [2] / no previously described/detected already published/assigned name are given.
